# Urinary cortisol is lower in pregnant women with higher pre-pregnancy BMI

**DOI:** 10.3389/fendo.2022.1014574

**Published:** 2023-01-11

**Authors:** Emily E. Hohman, Joshua M. Smyth, Katherine M. McNitt, Abigail M. Pauley, Danielle Symons Downs, Jennifer S. Savage

**Affiliations:** ^1^ Center for Childhood Obesity Research, University Park, PA, United States; ^2^ Department of Biobehavioral Health, Pennsylvania State University, University Park, PA, United States; ^3^ Department of Nutritional Sciences, Pennsylvania State University, University Park, PA, United States; ^4^ Department of Kinesiology, Pennsylvania State University, University Park, PA, United States; ^5^ Department of Obstetrics and Gynecology, Penn State College of Medicine, Hershey, PA, United States

**Keywords:** pregnancy, cortisol, stress, obesity, intensive longitudinal data

## Abstract

**Background/objectives:**

Although cortisol levels increase during normal pregnancy, particularly high levels of cortisol or stress have been associated with adverse maternal/child outcomes. Obesity is associated with altered cortisol metabolism, but there is limited information on pregnancy-related changes in cortisol in pregnant women with overweight/obesity. The objective of this study was to examine weekly measures of urinary cortisol and perceived stress throughout ~10-36 weeks gestation, if levels differ by pre-pregnancy BMI categories, and whether concurrent measures of urinary cortisol and perceived stress are associated.

**Methods:**

Longitudinal observational data from Healthy Mom Zone, a gestational weight management intervention, and an ancillary fetal growth study were combined. Pregnant women with normal (n=7), overweight (n=11), or obese (n=14) pre-pregnancy BMI were recruited at >8 weeks gestation. Overnight urinary cortisol and Perceived Stress Scale were measured weekly from ~10-36 weeks gestation.

**Results:**

Higher pre-pregnancy BMI was associated with overall lower urinary cortisol throughout gestation, but rate of increase in urinary cortisol across pregnancy was similar across weight status groups. Women with obesity reported higher levels of overall perceived stress than normal weight women. Regardless of weight status, perceived stress was not associated with gestational age or cortisol.

**Conclusions:**

Although women with obesity reported higher perceived stress, they had lower urinary cortisol than women with normal BMI, and gestation-related increases in cortisol were similar across weight groups and unrelated to perceived stress, suggesting that physiological factors that drive increases in cortisol as pregnancy may outweigh effects of stress and adiposity.

**Clinical trial registration:**

https://clinicaltrials.gov/ct2/show/NCT03945266, identifier (NCT03945266)

## 1 Introduction

Perceived psychosocial stress during pregnancy has been associated with a number of adverse pregnancy outcomes, including preterm birth and low birth weight (1). There is also evidence that prenatal maternal perceived stress may influence longer term outcomes for offspring health (2). Maternal level of cortisol, a biomarker of stress exposure, has separately been associated with a number of child outcomes, including fetal growth and infant cognitive ability, temperament, and stress regulation (3). Although it is known that cortisol increases during normal pregnancy (4), there is little information available on the trajectory of this change, as most studies examining changes in cortisol across pregnancy have measured cortisol at three (e.g. once per trimester) or fewer times throughout gestation (5–10). More frequent assessment and detailed examination of how cortisol level changes throughout pregnancy is needed to better understand the physiology of cortisol during pregnancy.

Over half of US women enter pregnancy with overweight or obesity (11), and pregnant women with obesity report greater levels of psychological distress (12). Maternal obesity is a well-established risk factor for adverse pregnancy outcomes, including gestational diabetes, caesarean birth, and large-for-gestational age birth (13), as well as long term obesity risk for children (14). In a rodent model, animals with a genetic predisposition to obesity were more susceptible to the effects of prenatal stress (15). In non-pregnant humans, perceived psychosocial stress has been associated with weight gain, particularly among those with an already elevated BMI (16). Furthermore, adults with greater abdominal adiposity have been shown to have greater cortisol reactivity to acute physical and psychosocial stressors (17). Obesity is also associated with alterations in cortisol metabolism (18), including placental metabolism (19). However, there has been little research on how the effects of prenatal perceived stress and cortisol may differ depending on maternal weight status.

The literature examining associations between cortisol levels in pregnancy and maternal weight status is scant; studies are either cross-sectional (20, 21) or longitudinal with a limited number of time points (8, 9, 22). These studies have consistently found lower levels of cortisol in pregnant women with obesity than those with normal weight, but whether patterns of change throughout pregnancy vary by weight status remains unclear. In addition, other factors such as maternal age (21–23), parity (21, 22, 24), and fetal sex (21, 25) have been inconsistently associated with maternal cortisol in the literature. Finally, although obesity-related differences in cortisol reactivity to stress have been reported in non-pregnant populations (17), studies examining concordance between cortisol and self-reported stress during pregnancy have not considered obesity as a moderator.

The objectives of this study were to a) describe how cortisol levels change throughout pregnancy using weekly urinary cortisol assessments, b) determine whether overall urinary cortisol level and it’s rate of change across gestation differ by pre-pregnancy BMI and demographic factors (i.e., maternal age, parity, fetal sex), and c) examine the association between urinary cortisol and self-reported perceived stress, and whether this relationship differs by pre-pregnancy BMI. Based on previous research (8, 10, 17, 20, 21), we hypothesized that a) urinary cortisol would increase across gestation; b) overall cortisol levels would be lower in women with pre-pregnancy overweight or obesity, older women, parous women, and women carrying male fetuses, and the increase in cortisol across gestation would be slower among women with pre-pregnancy overweight/obesity; and c) urinary cortisol would be associated with concurrent self-reported perceived stress, and pre-pregnancy BMI would moderate this association such that the magnitude of the association between cortisol and perceived stress would be greater among women with higher BMI.

## 2 Materials and methods

### 2.1 Participants

Data for this analysis were from two samples that were combined. Most participants (n=27) were from the Healthy Mom Zone (HMZ) study, a randomized-controlled trial designed to manage gestational weight gain in pregnant women with overweight or obesity (26). Women were eligible if they were >8 weeks pregnant with a single fetus, English-speaking, non-smoking, free of significant pregnancy complications or medical conditions, and had a BMI ranging from 24.5 to 45 kg/m^2^ (>40 kg/m^2^ with physician consultation). Exclusion criteria included diabetes at study entry, severe allergies or dietary restrictions, and contraindications to prenatal physical activity. Participants were randomized to a standard of care or the HMZ adaptive intervention, and all participants completed an intensive longitudinal data collection protocol, including an ancillary fetal growth study. Further details of the intervention and data collection procedures have been previously published (26). The remaining participants (n=5) included pregnant women with a BMI ≥ 18.5 enrolled into an observation only group to increase sample size for the fetal growth study and incorporate a greater BMI range. These women were not randomized to an intervention condition but completed the same measurement protocols as the participants enrolled in the HMZ study. With the exception of BMI, the same eligibility criteria were used as the HMZ study. This analysis includes a final sample of 32 women who were studied through approximately 36 weeks gestation. Demographics were reported at study enrollment, and fetal sex was abstracted from medical records. Height and pre-pregnancy weight were self-reported and used to calculate pre-pregnancy BMI. Gestational age was determined using last menstrual period. This project was completed in accordance with the Declaration of Helsinki. Written informed consent was obtained for each participant prior to randomization or completion of any study measures. Participants also provided consent for the study team to access their medical records. All procedures were approved by the Pennsylvania State University Institutional Review Board.

### 2.2 Urinary cortisol

Overnight urinary cortisol was measured once per week throughout the study. Urinary cortisol was used to reflect the systemic production of cortisol over a standardized period of time (versus, for example, salivary sampling that only reflects a point/momentary estimate) without the requirement of a blood draw. Women were asked to collect their urine from the time they went to bed at night through the first morning void. Because the prevalence and frequency of nighttime urination increases across gestation (27), this strategy helped ensure that samples reflected cortisol excretion over a consistent time window throughout gestation. Participants were instructed to refrigerate samples after collection, and following collection study staff retrieved and aliquoted the samples. Samples were frozen at -4°C until the end of the study when all samples were analyzed. Urinary cortisol was analyzed in duplicate using a competitive enzyme immunoassay (R&D Systems #KGE008B, Minneapolis, MN, assay range 0.2-10 ng/mL, sensitivity 0.111 ng/mL), and normalized to urinary creatinine (R&D Systems #KGE005, Minneapolis, MN, assay range 0.3-20 mg/dL, sensitivity 0.07 mg/dL). The inter-assay coefficient of variation was 14.0% for cortisol and 4.1% for creatinine, and the intra-assay coefficient of variation was 5.6% for cortisol and 2.5% for creatinine.

### 2.3 Perceived stress

Participants completed the Perceived Stress Scale (28) weekly as part of a paper survey that was turned in with each urine sample. This 10-item scale is a measure of the degree to which a person assesses situations in their life as stressful, and is a commonly used measure of global subjective psychosocial stress. Given the weekly sampling design, the questionnaire was modified to ask about the previous week rather than previous month. Cronbach’s alpha in this sample was 0.89, indicating very good internal consistency.

### 2.4 Statistical analysis

Descriptive statistics (mean ± SD or percentages) were generated for demographic variables. One single cortisol measurement was >15 SDs from the mean, and was thus excluded from the data set. Intraclass correlation coefficients (ICC) were calculated for urinary cortisol and perceived stress to determine the proportion of variance due to between-subjects variance. A series of simulations were used to estimate power to detect a BMI group x gestational age interaction. A set of 1000 simulated data sets were generated using variances and covariances from the current data set. Level 2 sample size was set to 32 individuals, and level 1 sample size set to 24 measurements per individual. Simulations indicated that there was 80% power to detect a BMI group by gestational age interaction with a difference in slopes of 0.08. The association between cortisol and predictor variables was evaluated using a generalized mixed-effects modeling approach (PROC GLIMMIX in SAS). All models utilized the Laplace estimation method and an unstructured covariance structure. Cortisol level across gestation was modeled using the loglinear distribution and identity link, with a random intercept and slope for gestational age in weeks. Linear, quadratic, and cubic fixed effects of gestational week were considered. Statistical significance of the fixed effects and Akaike information criterion (AIC) were used to determine that the linear model was the best fit, and a linear gestational week term was included in all subsequent models. Next, predictors including maternal pre-pregnancy BMI, age, parity, and fetal sex were examined in separate models. Pre-pregnancy BMI was analyzed both as a continuous variable to include maximal variability, and as a categorical variable, with participants classified as normal weight (BMI 18.5-24.9), overweight (BMI 25.0-29.9), or obese (BMI ≥30.0), for greater clinical interpretability. Main effects and interactions with gestational week were tested for each predictor. These analyses were repeated with perceived stress as the dependent variable. Finally, concurrent weekly perceived stress score was examined as a predictor of urinary cortisol. All models controlled for study group assignment (i.e. randomized to intervention, randomized to control, or non-randomized observation only). Due to variability in enrollment date and final study date, sample sizes at the earliest and latest gestational ages were small, so analyses were repeated including only data from 11-36 weeks gestation. Results were similar, so analyses with the full data set are included herein.

## 3 Results

Sample characteristics are described in **Table 1**. Participants (n=32) were predominantly non-Hispanic white (93.8%), married (90.6%), and pregnant with their first child (65.6%). The mean age of women at study entry was 30.5 ± 3.0 years and the mean pre-pregnancy BMI was 31.3 ± 7.1 kg/m^2^. Fourteen women (43.8%) were classified as having obesity (BMI ≥ 30), 11 (34.4%) were classified as having overweight (BMI 25-29.9), and 7 (21.9%) were classified as having a normal BMI (18.5-24.9). All women gave birth to live infants (56.3% male) with a mean birth weight of 3386 ± 619 grams and mean gestational age of 39.5 ± 1.4 weeks.

**Table 1:** Participant characteristics (n=32).

Variable	Mean (SD) or n (%)
Maternal age (years)	30.5 (3.0)
Pre-pregnancy BMI (kg/m^2^)	31.3 (7.1)
Gestational week at study entry	10.6 (1.7)
Infant birth weight (g)	3386 (619)
Gestational age at birth (weeks)	39.5 (1.4)
Race-ethnicity, n (%)	
Non-Hispanic white	30 (93.8)
Hispanic white	1 (3.1)
Asian	1 (3.1)
Marital status, n (%)	
Married	29 (90.6)
Single	2 (6.3)
Divorced	1 (3.1)
Parity, n (%)	
0	21 (65.6)
1	11 (34.3)
Pre-pregnancy BMI classification, n (%)	
Normal weight (BMI 18.5-24.9)	7 (21.9)
Overweight (BMI 25.0-29.9)	11 (34.4)
Obese (BMI ≥ 30)	14 (43.8)
Fetal sex, n (%)	
Male	18 (56.3)
Female	14 (43.8)

Adherence to the urine collection protocol was very high. On average, women collected 24.4 (SD 2.6, range 16-29) urine samples, reflecting a mean of 94.4% compliance with weekly sample collection. The ICC for urinary cortisol was 0.41, indicating both within- and between-person variability. As expected, urinary cortisol increased significantly across gestation (**Table 2**). Across the whole sample, mean urinary cortisol approximately doubled from the start of the second trimester at 14 weeks (66.4 ± 30.5 ng/mg creatinine) through 36 weeks (131.5 ± 84.8 ng/mg creatinine), with an average rate of increase of 3.1% per week.

**Table 2:** Predictors of maternal urinary cortisol (ng/mg creatinine) and perceived stress.

	Urinary cortisol (ng/mg creatinine)			Perceived stress		
Predictor	Model estimate	SE	p-value	Model estimate	SE	p-value
Gestational age (weeks)	0.0302	0.0036	<0.0001	0.0021	0.0023	0.36
Pre-pregnancy BMI (kg/m^2^)	-0.0156	0.0072	0.03	0.0185	0.0062	0.003
Maternal age (years)	0.0079	0.0019	0.67	-0.0209	0.0159	0.19
Nulliparous	0.0220	0.1189	0.85	0.2585	0.0947	0.007
Fetal sex – female	-0.0651	0.1074	0.54	0.1100	0.0961	0.25
Perceived stress	-0.0004	0.0028	0.90	–	–	–

Estimates are from generalized linear mixed models using a loglinear distribution and including random intercept and random gestational age slope. All models controlled for study group, and gestational week was included in models testing demographic characteristics and perceived stress as predictors. Est, fixed effect estimate; SE, standard error; BMI, body mass index.

**Figure 1:**
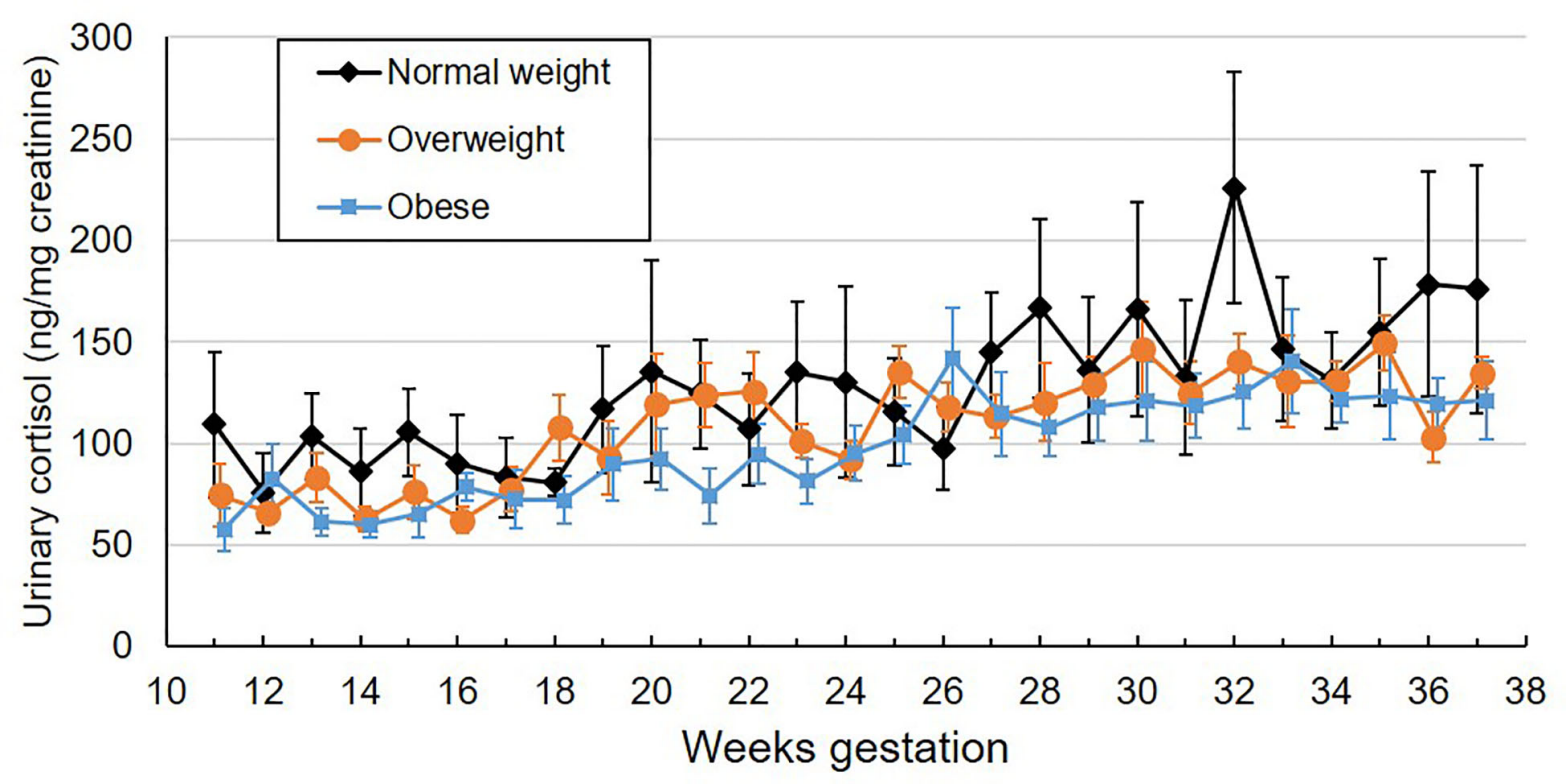
Mean weekly overnight urinary cortisol levels (ng/mg creatinine) across gestation by maternal pre-pregnancy BMI category. Values are mean ± standard error.

As a continuous variable, pre-pregnancy BMI was negatively associated with weekly urinary cortisol (**Table 2**), with a 1 unit increase in BMI being associated with -1.5% lower urinary cortisol. When considered as a categorical variable, women with overweight or obesity tended to have lower urinary cortisol than women with normal weight, though this difference did not meet the threshold for statistical significance (p=0.054). Mean values by BMI category across gestational week are plotted in **Figure 1**. On average, women with overweight/obesity had 28.2% lower urinary cortisol compared to women with normal weight. There was no significant interaction between pre-pregnancy BMI and gestational week, indicating that the rate of increase in urinary cortisol across pregnancy was similar across the range of BMI. There were no significant associations between urinary cortisol and maternal age, fetal sex, or parity, nor were there any interactions between gestational age and these factors.

**Figure 2:**
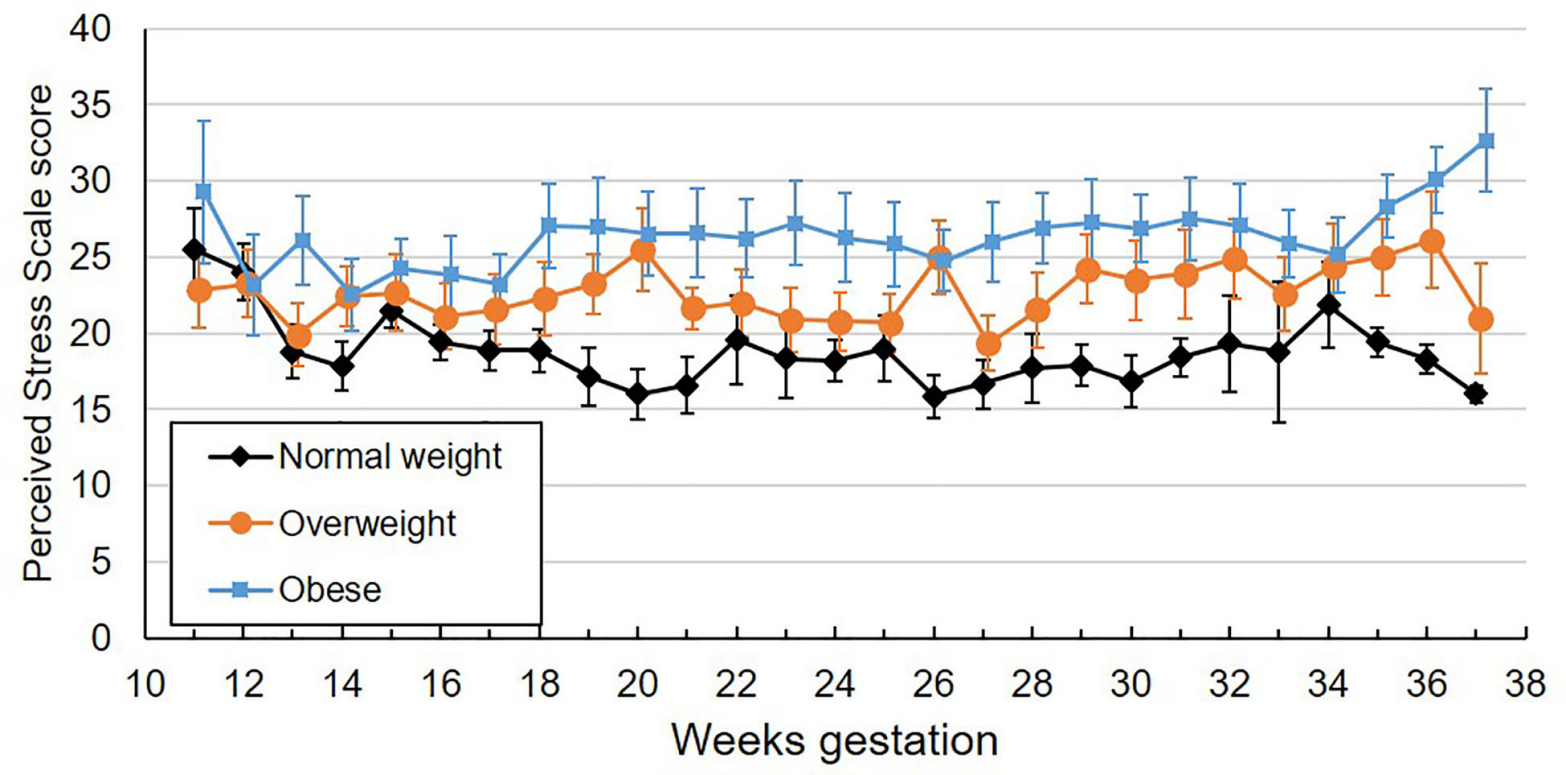
Mean weekly perceived stress scale scores across gestation by maternal pre-pregnancy BMI category. Values are mean ± standard error.

**Figure 3:**
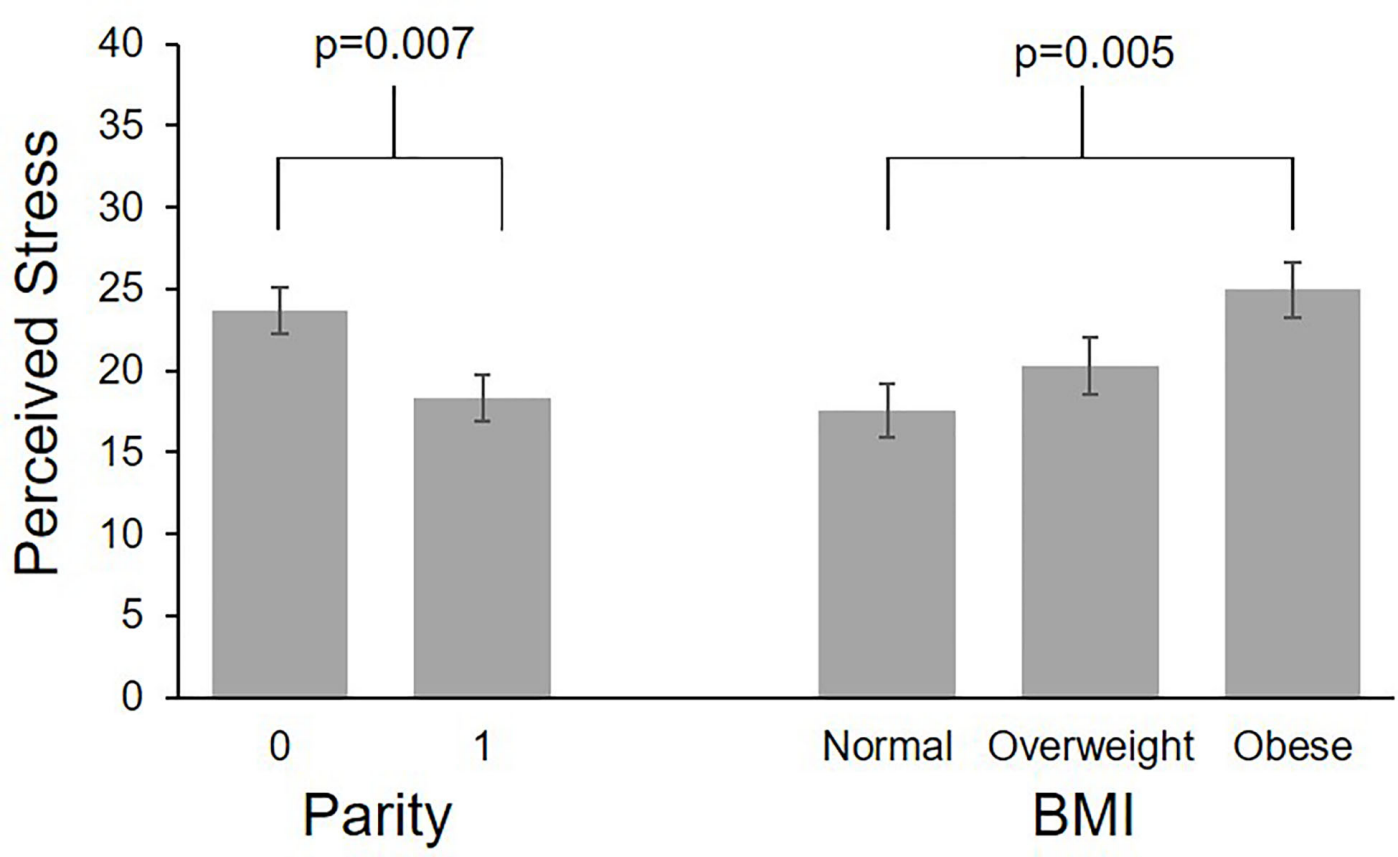
Differences in perceived stress by parity and pre-pregnancy BMI. Values are least square means ± standard error from multilevel models controlling for study group and gestational age.

Participants completed an average of 23.7 perceived stress scale questionnaires (SD 4.4, range 3-29), reflecting a mean compliance of 91.2% with the weekly questionnaires. One participant completed only 19% of their questionnaires; all others completed ≥ 80% of questionnaires. The ICC for perceived stress was 0.63. Self-reported perceived stress did not systematically change across gestation (**Table 2**). Mean values by BMI category across gestational week are plotted in **Figure 2**. Pre-pregnancy BMI was positively associated with perceived stress, with each 1 unit increase in BMI being associated with 1.9% greater perceived stress. When categorized into pre-pregnancy BMI classes, women with obesity had significantly higher perceived stress than women with normal BMI (p=0.005, **Figure 3**), with the average score among women with obesity being 42% higher than women with normal BMI. Women with pre-pregnancy overweight did not differ from either women with normal weight or obesity. Nulliparous women had 29.5% higher perceived stress scores than women with previous births (p=0.03, **Figure 3**). There was no association between perceived stress and either maternal age or fetal sex, and rate of change in perceived stress across pregnancy did not differ by these characteristics. There was also no significant association between cortisol and concurrent perceived stress in the sample as a whole (**Table 2**) or in any BMI group (pre-pregnancy BMI by perceived stress interaction, p=0.61).

## 4 Discussion

In this small but intensively characterized sample of pregnant women, we observed that urinary cortisol increased across gestation, and that levels were lower among women with higher pre-pregnancy BMI. Perceived stress was positively related to higher pre-pregnancy BMI. Unlike cortisol, perceived stress did not systematically change across gestation, and there was no association between urinary cortisol and perceived stress, regardless of BMI. These findings contribute to our understanding of perceived stress and cortisol physiology in pregnancies among women with obesity.

As expected, urinary cortisol increased across gestation, approximately doubling from late first trimester through ~36 weeks gestation. This is in line with previous studies which have reported a 1.5-3 fold increase in urinary cortisol during pregnancy (7, 10). Although the physiological role of the upregulation of cortisol during pregnancy is not fully understood, evidence suggests contributes to regulation of growth and the timing of birth (4). Although cortisol increased across gestation, there was no statistically significant, systematic change in perceived stress over time in this sample.

Also as hypothesized, we observed an inverse association between pre-pregnancy BMI and urinary cortisol, a finding consistent with previous research indicating that pregnant women with obesity have lower urinary (20), serum (8, 21), or salivary (8, 22) cortisol levels than those with lower BMIs. These findings in pregnancy are in contrast to studies in non-pregnant adults, which have mostly found either a positive or null relationship between BMI and cortisol (17). However, in contrast to our hypothesis, the rate of increase in cortisol across gestation did not differ by BMI. This finding differs from a previous report suggesting that women with obesity may not experience the same pregnancy-related increase in urinary cortisol excretion that is seen in women with normal BMI (8). Compared to this previous study, our sampling began earlier in pregnancy and was more frequent, which may have allowed us to better characterize gestational changes in urinary cortisol among women with obesity. However, our finding should be interpreted with caution as our study was not adequately powered to detect small-medium sized interaction effects. Despite lower cortisol levels, women with pre-pregnancy obesity reported greater levels of perceived stress than women with normal pre-pregnancy BMI. Compared to women without obesity, rates of depression and anxiety have been reported to be higher in pregnant women with obesity, who may experience unique sources of stress, such as weight-related stigma (12).

In contrast to our hypothesis, there was no association between urinary cortisol and perceived stress score; however, this is perhaps not surprising. The utility of cortisol as a biomarker of stress in pregnant women has been debated, as physiological changes in the regulation of the hypothalamic-pituitary-adrenal (HPA) axis that occur during pregnancy result in increased cortisol levels (4). In line with our findings, studies comparing cortisol and survey measures of stress during pregnancy have found little evidence of correlation between these measures (22, 29). However, studies using experimental protocols to induce stress responses (30) or experience sampling methods to measure subjective stress ‘in the moment’ (31) have found that cortisol remains responsive to stress during pregnancy. It is possible in our sample that more subtle fluctuations in cortisol due to within-person changes in weekly perceived stress were not detectible amid larger physiological changes related to advancing gestation. Future studies of the effects of stress during pregnancy would benefit from using a combination of different types of measures to assess stress levels.

We did not observe any of the hypothesized associations between cortisol levels and demographic factors including maternal age, parity, and fetal sex, although perceived stress was higher among nulliparous women. Previous studies have found higher levels of stress among younger pregnant women (32), older women experiencing their first pregnancy (32), and multiparous women (33), whereas nulliparous women may experience greater stress related to pregnancy-specific anxiety (34). Whether these relationships are reflected in cortisol levels is unclear. One cross-sectional study found that serum cortisol, assessed at an average of 12.9 weeks gestation, was higher among younger women (21). Studies examining cortisol later in gestation, however, have not found significant associations with maternal age (22, 23). Our sample lacked women at the extremes of child-bearing age range, which may have limited ability to detect such associations. The association between parity and cortisol level is also uncertain. Two studies have reported higher serum cortisol among nulliparous women (21, 24), while another found salivary cortisol to be higher in nulliparous women in early second trimester but not in later pregnancy (22). While fetal sex is likely unrelated to maternal exposure to stress, the effect of maternal stress on fetal programming of many outcomes is sex dependent (35), which could be related to differences in cortisol metabolism. One study found that women carrying female fetuses had higher serum cortisol levels (21). A longitudinal examination of salivary cortisol across the second half of pregnancy found that cortisol was higher in mothers of male fetuses from 24 to 30 week gestation, but mothers of female fetuses had higher levels after 30 weeks (25). Further research is needed to understand how cortisol metabolism and stress effects differ by fetal sex.

The intensive longitudinal characterization of cortisol and perceived stress across gestation is a strength of our study, but there are some limitations of note. The sample size was small and likely underpowered for analyses of interactions, and racially and socioeconomically homogenous, limiting generalizability. Maternal report of stress was collected using one instrument, the Perceived Stress Scale. Although this instrument is widely used and well-validated, it does not capture all types or sources of stress. Additional questions assessing pregnancy-specific stress would have strengthened the study.

In conclusion, this study demonstrated that pregnant women with overweight and obesity had consistently lower urinary cortisol than women with normal pre-pregnancy BMI across the study period of ~10-36 weeks gestation. The rate of increase in cortisol across gestation, however, was similar across BMI category, in turn suggesting that the physiological upregulation of cortisol that occurs as gestation advances may be common feature of pregnancy across all weight statuses. This finding, coupled with the fact that studies in non-pregnant individuals tend to observe positive or null associations between adiposity and cortisol, suggests that obesity-related differences in maternal cortisol may arise early in gestation. Further research is needed to evaluate the mechanisms and consequences of obesity-related alterations of cortisol metabolism in early pregnancy. Regardless of BMI status, weekly reports of subjective stress throughout gestation were not predictive of concurrent weekly urinary cortisol levels in this sample. Longitudinal studies of pregnancy with intensively collected data using a combination of biomarkers (e.g. cortisol), ambulatory assessment (e.g. heart rate monitoring), and subjective measures of stress (e.g. ecological momentary assessment, pregnancy specific stress) would help to determine if stress management is a viable intervention target to optimize maternal and fetal outcomes.

## Data availability statement

The raw data supporting the conclusions of this article will be made available by the authors, without undue reservation.

## Ethics statement

The studies involving human participants were reviewed and approved by Pennsylvania State University Institutional Review Board. The patients/participants provided their written informed consent to participate in this study.

## Author contributions

EH contributed to study design, analysed and interpreted the data, and drafted the manuscript. JS contributed to study design, data interpretation, and critical revision of the manuscript. KM and AP contributed to study design, data collection, and critical revision of the manuscript. DS and JS lead the study design, contributed to data interpretation, and critical revision of the manuscript. All authors contributed to the article and approved the submitted version.

## References

[1] Hobel CJ Goldstein A Barrett ES. Psychosocial stress and pregnancy outcome. Clin Obstet Gynecol. (2008) 51(2):333–48. doi: 10.1097/GRF.0b013e31816f2709 18463464

[2] Van den Bergh BRH van den Heuvel MI Lahti M Braeken M de Rooij SR Entringer S. Prenatal developmental origins of behavior and mental health: The influence of maternal stress in pregnancy. Neurosci Biobehav Rev (2017) 117:26ߝ64. doi: 10.1016/j.neubiorev.2017.07.003 28757456

[3] Zijlmans MA Riksen-Walraven JM de Weerth C. Associations between maternal prenatal cortisol concentrations and child outcomes: A systematic review. Neurosci Biobehav Rev (2015) 53:1–24. doi: 10.1016/j.neubiorev.2015.02.015 25795521

[4] Duthie L Reynolds RM. Changes in the maternal hypothalamic-pituitary-adrenal axis in pregnancy and postpartum: influences on maternal and fetal outcomes. Neuroendocrinology (2013) 98(2):106–15. doi: 10.1159/000354702 23969897

[5] Conde A Figueiredo B. 24-h urinary free cortisol from mid-pregnancy to 3-months postpartum: gender and parity differences and effects. Psychoneuroendocrinology (2014) 50:264–73. doi: 10.1016/j.psyneuen.2014.08.013 25247747

[6] Mikkelsen AL Felding C Hasselbalch H. Urinary free cortisol during pregnancy. Acta Obstet Gynecol Scand (1984) 63(3):253–6. doi: 10.3109/00016348409155508 6730942

[7] Cousins L Rigg L Hollingsworth D Meis P Halberg F Brink G. Qualitative and quantitative assessment of the circadian rhythm of cortisol in pregnancy. Am J Obstet Gynecol. (1983) 145(4):411–6. doi: 10.1016/0002-9378(83)90309-5 6297302

[8] Stirrat LI O’Reilly JR Barr SM Andrew R Riley SC Howie AF. Decreased maternal hypothalamic-pituitary-adrenal axis activity in very severely obese pregnancy: Associations with birthweight and gestation at delivery. Psychoneuroendocrinology (2016) 63:135–43. doi: 10.1016/j.psyneuen.2015.09.019 26444587

[9] Aubuchon-Endsley NL Bublitz MH Stroud LR. Pre-pregnancy obesity and maternal circadian cortisol regulation: Moderation by gestational weight gain. Biol Psychol (2014) 102:38–43. doi: 10.1016/j.biopsycho.2014.07.006 25038305 PMC4157070

[10] Jung C Ho JT Torpy DJ Rogers A Doogue M Lewis JG. A longitudinal study of plasma and urinary cortisol in pregnancy and postpartum. J Clin Endocrinol Metab (2011) 96(5):1533–40. doi: 10.1210/jc.2010-2395 21367926

[11] Branum AM Kirmeyer SE Gregory EC. Prepregnancy body mass index by maternal characteristics and state: Data from the birth certificate, 2014. Natl Vital Stat Rep (2016) 65(6):1–11. 27508894

[12] Faria-Schutzer DB Surita FG Nascimento SL Vieira CM Turato E. Psychological issues facing obese pregnant women: a systematic review. J Matern Fetal Neonatal Med (2017) 30(1):88–95. doi: 10.3109/14767058.2016.1163543 26952707

[13] Marchi J Berg M Dencker A Olander EK Begley C. Risks associated with obesity in pregnancy, for the mother and baby: a systematic review of reviews. Obes Rev (2015) 16(8):621–38. doi: 10.1111/obr.12288 26016557

[14] Yu Z Han S Zhu J Sun X Ji C Guo X. Pre-pregnancy body mass index in relation to infant birth weight and offspring overweight/obesity: a systematic review and meta-analysis. PloS One (2013) 8(4):e61627. doi: 10.1371/journal.pone.0061627 23613888 PMC3628788

[15] Balasubramanian P Varde PA Abdallah SL Najjar SM MohanKumar PS MohanKumar SM. Differential effects of prenatal stress on metabolic programming in diet-induced obese and dietary-resistant rats. Am J Physiol Endocrinol Metab (2015) 309(6):E582–8. doi: 10.1152/ajpendo.00167.2015 PMC4572454 26219866

[16] Block JP He Y Zaslavsky AM Ding L Ayanian JZ. Psychosocial stress and change in weight among US adults. Am J Epidemiol. (2009) 170(2):181–92. doi: 10.1093/aje/kwp104 PMC2727271 19465744

[17] Rodriguez ACI Epel ES White ML Standen EC Seckl JR Tomiyama AJ. Hypothalamic-pituitary-adrenal axis dysregulation and cortisol activity in obesity: A systematic review. Psychoneuroendocrinology (2015) 62:301–18. doi: 10.1016/j.psyneuen.2015.08.014 26356039

[18] Andrew R Phillips DI Walker BR. Obesity and gender influence cortisol secretion and metabolism in man. J Clin Endocrinol Metab (1998) 83(5):1806–9. doi: 10.1210/jcem.83.5.4951 9589697

[19] Johns EC Denison FC Reynolds RM. The impact of maternal obesity in pregnancy on placental glucocorticoid and macronutrient transport and metabolism. Biochim Biophys Acta Mol Basis Dis (2019) 1866:165374. doi: 10.1016/j.bbadis.2018.12.025 30684643

[20] Luiza JW Gallaher MJ Powers RW. Urinary cortisol and depression in early pregnancy: role of adiposity and race. BMC Pregnancy Childbirth. (2015) 15:30. doi: 10.1186/s12884-015-0466-7 25885329 PMC4335360

[21] Bleker LS Roseboom TJ Vrijkotte TG Reynolds RM de Rooij SR. Determinants of cortisol during pregnancy - the ABCD cohort. Psychoneuroendocrinology (2017) 83:172–81. doi: 10.1016/j.psyneuen.2017.05.026 28641158

[22] Harville EW Savitz DA Dole N Herring AH Thorp JM. Stress questionnaires and stress biomarkers during pregnancy. J Womens Health (Larchmt). (2009) 18(9):1425–33. doi: 10.1089/jwh.2008.1102 PMC2825685 19757520

[23] Garcia-Blanco A Monferrer A Grimaldos J Hervas D Balanza-Martinez V Diago V. A preliminary study to assess the impact of maternal age on stress-related variables in healthy nulliparous women. Psychoneuroendocrinology (2017) 78:97–104. doi: 10.1016/j.psyneuen.2017.01.018 28187401

[24] Gillespie SL Mitchell AM Kowalsky JM Christian LM. Maternal parity and perinatal cortisol adaptation: The role of pregnancy-specific distress and implications for postpartum mood. Psychoneuroendocrinology (2018) 97:86–93. doi: 10.1016/j.psyneuen.2018.07.008 30015009 PMC6582962

[25] DiPietro JA Costigan KA Kivlighan KT Chen P Laudenslager ML. Maternal salivary cortisol differs by fetal sex during the second half of pregnancy. Psychoneuroendocrinology (2011) 36(4):588–91. doi: 10.1016/j.psyneuen.2010.09.005 PMC3021768 20940089

[26] Symons Downs D Savage JS Rivera DE Smyth JM Rolls BJ Hohman EE. Individually tailored, adaptive intervention to manage gestational weight gain: Protocol for a randomized controlled trial in women with overweight and obesity. JMIR Res Protoc (2018) 7(6):e150. doi: 10.2196/resprot.9220 29884603 PMC6015270

[27] FitzGerald MP Graziano S. Anatomic and functional changes of the lower urinary tract during pregnancy. Urol Clin North Am (2007) 34(1):7–12. doi: 10.1016/j.ucl.2006.10.007 17145355

[28] Cohen S Kamarck T Mermelstein R. A global measure of perceived stress. J Health Soc Behav (1983) 24(4):385–96. doi: 10.2307/2136404 6668417

[29] Rothenberger SE Moehler E Reck C Resch F. Prenatal stress: course and interrelation of emotional and physiological stress measures. Psychopathology (2011) 44(1):60–7. doi: 10.1159/000319309 21072001

[30] De Weerth C Wied GD Jansen LM Buitelaar JK. Cardiovascular and cortisol responses to a psychological stressor during pregnancy. Acta Obstet Gynecol Scand (2007) 86(10):1181–92. doi: 10.1080/00016340701547442 17851798

[31] Giesbrecht GF Campbell T Letourneau N Kaplan BJ Team APS. Advancing gestation does not attenuate biobehavioural coherence between psychological distress and cortisol. Biol Psychol (2013) 93(1):45–51. doi: 10.1016/j.biopsycho.2013.01.019 23410761

[32] Aasheim V Waldenstrom U Hjelmstedt A Rasmussen S Pettersson H Schytt E. Associations between advanced maternal age and psychological distress in primiparous women, from early pregnancy to 18 months postpartum. BJOG (2012) 119(9):1108–16. doi: 10.1111/j.1471-0528.2012.03411.x 22703587

[33] Dipietro JA Costigan KA Sipsma HL. Continuity in self-report measures of maternal anxiety, stress, and depressive symptoms from pregnancy through two years postpartum. J Psychosom Obstet Gynaecol. (2008) 29(2):115–24. doi: 10.1080/01674820701701546 PMC9566577 18655259

[34] Dunkel Schetter C Niles AN Guardino CM Khaled M Kramer MS. Demographic, medical, and psychosocial predictors of pregnancy anxiety. Paediatr Perinat Epidemiol. (2016) 30(5):421–9. doi: 10.1111/ppe.12300 27221458

[35] Hicks LM Swales DA Garcia SE Driver C Davis EP. Does prenatal maternal distress contribute to sex differences in child psychopathology? Curr Psychiatry Rep (2019) 21(2):7. doi: 10.1007/s11920-019-0992-5 30729361

